# Prenatal Diagnosis of ACTG2-Related Megacystis–Microcolon–Intestinal Hypoperistalsis Syndrome—Case Report and Systematic Review

**DOI:** 10.3390/jcm14093204

**Published:** 2025-05-06

**Authors:** Neha Ravi, Sailesh Kumar, Aparna Ramachandran

**Affiliations:** 1 Centre for Maternal and Fetal Medicine, Mater Mothers’ Hospital, Brisbane 4101, Australia; 2 Mater Research Institute, The University of Queensland, Brisbane 4101, Australia; 3 School of Medicine, The University of Queensland, Brisbane 4072, Australia

**Keywords:** megacystis, Berdon syndrome, prenatal diagnosis, ACTG2 mutation

## Abstract

**Background/Objectives**: Megacystis–microcolon–intestinal hypoperistalsis syndrome (MMIHS) is characterized by smooth muscle dysfunction and results in severe bladder dilatation and intestinal dysmotility. Prenatal diagnosis is challenging due to the non-specific nature of ultrasound findings and the limitations of current genetic testing. We present a case of persistent fetal megacystis, with genetic testing confirming MMIHS, and a systematic review of prenatally diagnosed cases. **Methods**: An electronic search of the PubMed, Medline, Web of Science and CORE databases was conducted to identify reports of genetic prenatal diagnoses of MMIHS. The inclusion criteria were cases of prenatally suspected MMIHS with a genetic diagnosis based on prenatal samples. Reports that described neonatal or paediatric cases or lacked clinical details or genetic testing results were excluded, and the clinical details for the included cases were collected. **Results**: We identified six publications describing 17 cases of MMIHS confirmed on genetic testing. Including our case, 18 cases are described in this manuscript. Most cases (72.2%) presented in the second or third trimester of pregnancy; the majority (55.6%) were due to ACTG2 mutations. All cases had fetal megacystis detected on ultrasound. Five cases (27.8%) also had a known family history of MMIHS. The majority of the cases (77.8%) resulted in the termination of pregnancy. **Conclusions**: MMIHS is a rare condition with significant morbidity and mortality and prenatal diagnosis remains challenging. ACTG2 mutations are described in over half of these cases. These data contribute to the limited literature on its prenatal presentation and the evolving role of prenatal molecular genetic testing.

## 1. Introduction

Megacystis–microcolon–intestinal hypoperistalsis syndrome (MMIHS) (also known as Berdon syndrome) is a rare, sporadic congenital disorder. It is more prevalent in females, although the reason for this is unclear, and is characterized by smooth muscle dysfunction resulting in severe bladder dilatation, intestinal dysmotility and hypoplastic colon [1,2,3]. The clinical outcomes have historically been poor, mainly because of intestinal-related complications with high rates of malnutrition, liver failure, complications from parenteral feeding, short bowel syndrome and functional bowel obstruction. These individuals are also at risk of frequent episodes of urosepsis secondary to chronic bladder dysfunction [1,2]. More recently, advances in intestinal transplantation and immunosuppression have improved survival for affected individuals [2].

Definitive prenatal diagnosis of MMIHS remains challenging. There are no specific prenatal ultrasound features pathognomonic of the diagnosis. MMIHS should be considered whenever there is significant and persistent bladder dilatation. In contrast to megacystis due to posterior urethral valves or urethral atresia, the enlarged bladder seen in MMIHS is hypotonic rather than obstructed. Renal pelvis dilatation is often present and dilated loops of bowel and/or a dilated stomach may be evident. The amniotic fluid volume is frequently normal, although polyhydramnios has also been described in some cases [4].

The most common genetic causes of MMIHS are mutations in ACTG2, which account for 44% of all cases and arise either de novo or inherited in an autosomal dominant fashion [1,3]. A number of autosomal recessive mutations have also been described [1,5,6,7]. However, up to half of all cases of MMIH do not have an identified genetic mutation [1], although this may change in the future with evolving genetic techniques.

MMIHS is a lifelong condition, with traditionally limited treatment options and overall poor prognosis; as such, prenatal counselling in suspected cases typically includes discussion of active treatment options or pregnancy termination.

We describe a case of prenatally suspected MMIHS based on ultrasound abnormalities and subsequently confirmed on whole-exome sequencing. Written informed consent was obtained from the patient for the publication of this case report. We also performed a systematic review to describe key issues in the prenatal diagnosis of MMIHS due to the rarity of the condition.

## 2. Materials and Methods

**Figure 1:**
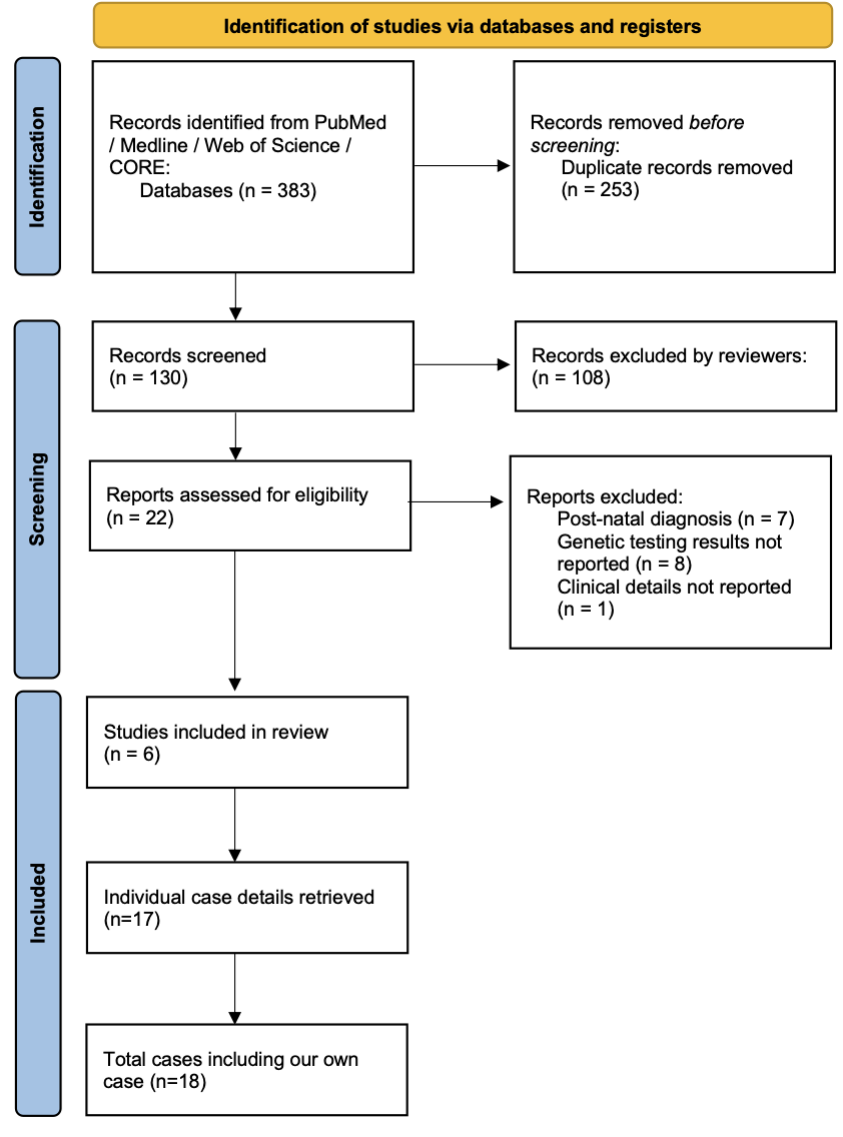
PRISMA flowchart describing search strategy.

An electronic search was conducted of the PubMed, Medline, Web of Science and COnnecting Repositories (CORE) databases in February 2025 to identify case reports of prenatal diagnosis of MMIHS. These databases were chosen to ensure coverage of conventional published literature and grey literature sources. The search strategy was not limited to a certain date, type of publication or language. Search terms were a combination of ‘prenatal’ and ‘MMIHS’, ‘MMIH’, ‘megacystis microcolon’, ‘Berdon’ or ‘visceral myopathy’; no other synonym truncations were employed. Inclusion criteria were cases of prenatally suspected MMIHS with a genetic diagnosis based on prenatal samples. Reports that described neonatal or paediatric cases or lacked genetic information were excluded. The PRISMA search flowchart is provided in Figure 1; the review was not registered. Data were collected about diagnostic information (gestational age, ultrasound features, presence of additional structural abnormalities), genetic testing information (type of testing, genetic test results) and pregnancy outcome. Two reviewers (NR and AR) screened 22 full-text publications and 6 reports [5,8,9,10,11,12] were ultimately included. Any disagreements about the included reports were resolved after discussion with all authors.

## 3. Results

### 3.1. Case Report

A 29-year-old multiparous woman was referred for tertiary evaluation at 21 weeks’ gestation because of an enlarged fetal bladder. She had had one previous uncomplicated pregnancy resulting in a vaginal delivery at term of a 4.2 kg female infant. The current pregnancy was naturally conceived, and routine non-invasive prenatal screening in the first trimester reported a low risk for trisomies 21, 18, and 13 and sex chromosome aneuploidies.

Detailed ultrasound at 21 weeks showed a male fetus with a markedly dilated bladder that did not empty and distending the fetal abdomen. Both kidneys appeared normal, as was the amniotic fluid volume. The initial working diagnosis was of partial lower urinary tract obstruction secondary to posterior urethral valves as the most common cause. The parents were counselled about the ultrasound findings, possible diagnosis and outcomes. Repeat ultrasound scans at 24 weeks’ and 28 weeks’ gestation showed persistent megacystis, mild bilateral renal pelvis dilatation measuring 8 mm bilaterally and normal amniotic fluid volume. There was no ultrasound evidence of dysplastic changes in the kidneys.

A follow-up ultrasound scan at 32 weeks revealed a large-for-gestational-age fetus (estimated fetal weight 96th centile for gestational age with abdominal circumference > 99th centile) with a massively enlarged bladder (720 mL in volume) and polyhydramnios (deepest pocket 11 cm). The differential diagnosis for polyhydramnios includes intestinal obstruction and maternal diabetes; however, the fetal stomach was visualised and maternal oral glucose tolerance test screen for gestational diabetes mellitus was normal. No other structural abnormalities were seen.

Because of the polyhydramnios, which is not associated with lower urinary tract obstruction, further investigation was indicated. Amnioreduction for maternal symptom management and concurrent vesicocentesis to assess bladder refilling were performed at 33 weeks’ gestation. Amniotic fluid was sent for fluorescent in-situ hybridisation and microarray, and trio whole-exome sequencing was also undertaken. The bladder was noted to refill close to its pre-vesicocentesis size 24 h later, indicating ongoing fetal urine production. Chromosome microarray analysis from the amniotic fluid revealed no abnormalities. Trio whole-exome sequencing results identified a de novo heterozygous pathogenic mutation in the ACTG2 gene consistent with a diagnosis of MMIHS. After multidisciplinary consultation involving neonatology, paediatric nephrology and paediatric surgery specialists and a discussion regarding both the short- and long-term outcomes and quality of life considerations, the parents elected for the termination of the pregnancy at 35 weeks’ gestation. After intracardiac potassium chloride feticide, repeat vesicocentesis for bladder decompression and external cephalic version, vaginal birth of a stillborn male infant weighing 3860 g ensued following the induction of labour.

Postmortem examination confirmed an enlarged, thin-walled bladder and mild bilateral renal cortical thinning. There were no macroscopic intestinal abnormalities. Placental histopathology was unremarkable.

### 3.2. Systematic Review

We identified 6 publications reporting outcomes of 17 cases of MMIHS with genetic information. The details of these 17 cases and our case are included in Table 1. Most of the reported cases (14/18, 72.2%) presented in the second or third trimester of pregnancy. All cases were suspected due to ultrasound features: the most common ultrasound finding was megacystis (100%); five cases (27.8%) had additional urinary tract abnormalities (renal pelvis dilatation, hydronephrosis or thinning of renal cortex). Only two cases (11.1%) had obvious sonographic bowel abnormalities. In three cases (16.7%), there were additional structural abnormalities. The majority of the cases (14/18, 77.8%) resulted in termination of pregnancy. There were two (11.1%) livebirths and two (11.1%) intrauterine fetal deaths.

The majority of prenatally diagnosed cases (10/18, 55.6%) were due to ACTG2 mutations. Five cases (27.8%) were known to have a family history of MMIHS at the time of prenatal testing.

## 4. Discussion

In this report, we detail a case of MMIHS with a typical causative genetic mutation and the results of a systematic review of this condition. The clinical features of our case developed in the second half of pregnancy and evolved with time, emphasizing the challenge in making an early diagnosis and the importance of ongoing maternal–fetal medicine review. Our results demonstrate the rarity of this condition and the challenges of a definitive prenatal diagnosis—only 20% of cases are reliably diagnosed before birth [4,13]. Historically, most cases were diagnosed after birth either based on clinical features or after postmortem. The true prevalence is thus likely to be underestimated because many pregnancies are terminated before the diagnosis is confirmed [4,10]. Additionally, estimations of prevalence based on the published literature are also likely to be inaccurate due to publication bias, with positive prenatal or genetic findings more likely to be reported; ascertainment bias is also an issue, as not all cases may undergo full investigation including genetic testing, and cases with a positive family history are more likely to be recognised. Our systematic review also demonstrates the rapid evolution of targeted genetic testing for MMIHS: the first report of ACTG2-associated MMIHS was published in 2014 [3], with subsequent publications detailing new pathogenic mutations and increased rates of prenatal diagnosis of MMIHS. Future reports should prioritise phenotype–genotype correlation to improve case recognition and diagnostic yield for this rare condition.

Our systematic review also describes the typical but varied prenatal ultrasound features of MMIHS. We show that urinary tract abnormalities predominate, with all cases manifesting with megacystis and variable degrees of renal pelvis dilatation. While the gastrointestinal manifestations of MMIHS lead to significant morbidity postnatally and in adult life due to hypomotility and malabsorption [2], gastrointestinal tract abnormalities are rarely seen on prenatal ultrasound. Our case was notable because of the severity of the polyhydramnios. We speculate that this may have resulted from gastrointestinal hypomotility because no other possible causes were found following maternal investigations nor on postmortem examination. Both polyhydramnios and a normal amniotic fluid volume have previously been associated with MMIHS in the prenatal setting [4]. Additionally, the postmortem examination in our case did not reveal any macroscopic bowel abnormalities. Some previous reports have shown either a normal or small calibre of the intestines [14,15]. Lastly, the pathophysiology of MMIHS is incompletely understood, with biopsy specimens of the bowel often showing normal submucosal ganglionic cells [14,15]; there is currently inadequate information to link particular phenotypes with specific genetic mutations. This variability in postmortem findings may also limit the recognition of MMIHS and should be taken into account when recommending further genetic investigation.

In our systematic review, it was unclear how many cases had confirmatory prenatal genetic testing results available during pregnancy. Similarly to our patient, the majority of the cases (72.2%) in our review were suspected based on ultrasound features in the second or third trimesters. In many cases, including our own (11/18, 61.1%), the decision for the termination of pregnancy was made because of the severity of the ultrasound findings. This likely reflects the current availability and workflows of molecular genetic testing, and, possibly, jurisdictional gestational limits on terminations of pregnancies. An improved ability to diagnose MMIH in the prenatal setting in the future would help to encourage multidisciplinary counselling, particularly regarding advances in care for MMIH, and allow families to make informed decisions.

Regardless of the availability of targeted genetic testing, MMIHS is still likely to be underdiagnosed prenatally, as cases with milder phenotypes are less likely to be tested, in both the fetal and postnatal settings. In our review, we identified three cases of familial ACTG2-related MMIHS; one case was associated with somatic mosaicism in an asymptomatic parent [11] and in two other cases, a parent was not initially known to be affected but was diagnosed after the index pregnancy, and found to have a milder phenotype [8,10]. This also highlights the difficulty in prenatal counselling regarding outcomes for rare conditions.

Our understanding of genetic variants causing MMIHS is currently limited but continuing to develop as more cases are tested [3,5]. Additionally, the yield of testing is also dependent on the type of test chosen. ACTG2 mutations are the most common MMIHS-associated mutation: our systematic review found that ACTG2 mutations are present in 55% of prenatally diagnosed cases. Other series have shown that ACTG2 mutations account for 44% of cases of MMIHS [1]. As ACTG2 is not currently included in all available congenital anomalies of the kidney and urinary tract ‘panel’ tests [16], it is likely that many cases with a similar phenotype are missed, even on panel testing. Conversely, Saraiva et al. [17] describe two cases of suspected MMIHS where either targeted ACTG2 testing or whole-exome sequencing were uninformative. However, the ability to store DNA may allow for currently non-informative testing to be revisited in the future with detection of potentially causative mutations. This is evidenced by a recent report by Billon et al. [5] describing genetic testing of stored fetal DNA samples after termination of pregnancy, often when postmortem findings were either uninformative or equivocal. They detected cases of ACTG2 mutation as well as the discovery of a new potential candidate gene, PDCL3. Although it is known that other mutations are involved in this condition (Table 2) it is likely that more will be discovered in the future. This emphasizes the rapidly evolving nature of genetic testing options for complex prenatal cases, and the need to offer genetic testing widely. In addition to prognostication, accurate prenatal testing will improve active early postnatal care, and may also develop avenues for prenatal genetic therapies in the future.

We acknowledge that access to expanded genetic testing is not universal, and that it is currently difficult to apply the findings of our systematic review to low-resource healthcare settings due to the current cost of testing. However, improved prenatal detection of MMIHS may also help to develop alternative lower-cost diagnostic pathways in the future.

## 5. Conclusions

This case report and systematic review of prenatally diagnosed cases of MMIHS underscore the importance of integrating advances in prenatal imaging, molecular genetic testing and multidisciplinary input in the diagnosis and management of rare congenital disorders such as MMIHS. The identification of an enlarged fetal bladder and subsequent trio whole-exome sequencing led to a confirmed diagnosis of a de novo pathogenic variant in the ACTG2 gene, providing a definitive diagnosis and enabling parental counselling about the likelihood of recurrence. As MMIHS is a rare condition with significant morbidity and mortality, our case and accompanying systematic review contributes to the limited literature about this condition and the evolving role of prenatal molecular genetic testing. Future research should focus on improved case finding with wider access to expanded genetic testing to further our understanding of this underreported condition, and on improving diagnostic pathways in low-resource settings.

## Figures and Tables

**Table 1:** Cases of MMIHS with prenatal diagnosis.

Authors	Year	GA at Testing	Trigger for Testing	Ultrasound Features	Type of Genetic Testing	MMIH Gene Result	Outcome
Milunsky [11]	2017	22	US findings	Megacystis, hydronephrosis, thinning of renal cortex	Targeted	ACTG2	TOP GA 22
Wang [9]	2019	13	US findings	Megacystis, oligohydramnios	Trio WES	MYH11	TOP GA 17
Wang [9]	2019	14	US findings	Megacystis	Trio WES	MYH11	TOP GA 16
Markota [12]	2020	25	US findings	Megacystis, prominent small bowel	Targeted	ACTG2	Livebirth at GA 34 (preterm labour)
Billon [5]	2020	21	US findings	Megacystis	Targeted	ACTG2	TOP GA 24
Billon [5]	2020	19	US findings	Megacystis	Targeted	ACTG2	TOP GA 25
Billon [5]	2020	20	US findings	Megacystis, echogenic bowel, omphalocele	Targeted	ACTG2	TOP GA 29
Billon [5]	2020	16	US findings	Megacystis	Trio WES	MYH11	TOP GA 16
Billon [5]	2020	Unknown	US findings + family history	Megacystis	Trio WES	MYH11	IUFD GA 28
Billon [5]	2020	10	US findings + family history	Megacystis	Trio WES	MYH11	TOP GA 10
Billon [5]	2020	15	US findings + family history	Megacystis	Trio WES	MYL9	TOP GA 27
Billon [5]	2020	Second trimester	US findings	Megacystis, bilateral pelvicalyceal dilatation, single umbilical artery	Trio WES	PDCL3	TOP GA 30
Billon [5]	2020	12	US findings + family history	Megacystis, bilateral diaphragmatic hernia	Trio WES	PDCL3	IUFD GA 12
Krabek [8]	2023	27	US findings + family history	Megacystis, mild pyelectasis	Trio WES	ACTG2	Livebirth at term
Yu [10]	2024	23	US findings	Megacystis, mild pyelectasis	Trio WES	ACTG2	TOP GA 23
Yu [10]	2024	17	US findings	Megacystis	Trio WES	ACTG2	TOP GA 23
Yu [10]	2024	22	US findings	Megacystis	Trio WES	ACTG2	TOP GA 22
Ravi	2025	21	US findings	Megacystis, bilateral renal pelvis dilatation, polyhydramnios	Trio WES	ACTG2	TOP GA 35

US = ultrasound; WES = whole-exome sequencing; TOP = termination of pregnancy; GA = gestational age; IUFD = intrauterine fetal death.

**Table 2:** Currently known genetic causes of MMIHS.

Gene/Locus	Location	Inheritance
ACTG2	2p13.1	AD
MYLK	3q21.1	AR
MYL9	20q11.23	AR
MYH11	16p13.11	AR
LMOD1	1q32.1	AR

AD = autosomal dominant; AR = autosomal recessive.

## Data Availability

Data can be made available upon reasonable request and with the patient’s consent.

## Abbreviations

The following abbreviations are used in this manuscript:

MMIHS	Megacystis–microcolon–intestinal hypoperistalsis syndrome
US	Ultrasound
WES	Whole-exome sequencing
TOP	Termination of pregnancy
GA	Gestational age
IUFD	Intrauterine fetal death
AD	Autosomal dominant
AR	Autosomal recessive
